# Genome-wide identification of the *sorghum* OVATE gene family and revelation of its expression characteristics in *sorghum* seeds and leaves

**DOI:** 10.1038/s41598-024-66103-z

**Published:** 2024-07-02

**Authors:** Yanlin An, Xiaobo Xia, Xiaoqin Zhang, Li Liu, Sixia Jiang, Tingting Jing, Feng Zhang

**Affiliations:** 1 Department of Food Science and Engineering, Moutai Institute, Renhuai, China; 2 State Key Laboratory of Tea Plant Biology and Utilization, Anhui Agricultural University, Hefei, China; 3 College of Plant Protection, Nanjing Agricultural University, Nanjing, 210095 China

**Keywords:** *Sorghum*, OVATE, Gene family, Transcriptome sequencing, Agricultural genetics, Gene expression

## Abstract

The OVATE gene family plays an important role in regulating the development of plant organs and resisting stress, but its expression characteristics and functions in *sorghum* have not been revealed. In this study, we identified 26 OVATE genes in the *sorghum* BTx623 genome, which were divided into four groups and distributed unevenly across 9 chromosomes. Evolutionary analysis showed that after differentiation between *sorghum* and *Arabidopsis*, the OVATE gene family may have experienced unique expansion events, and all OVATE family members were negatively selected. Transcriptome sequencing and RT-qPCR results showed that OVATE genes in *sorghum* showed diverse expression characteristics, such as gene *SORBl_3001G468900* and *SORBl_3009G173400* were significantly expressed in seeds, while *SORBI_3005G042700* and *SORBI_3002G417700* were only highly expressed in L1. Meantime, in the promoter region, a large number of hormone-associated cis-acting elements were identified, and these results suggest that members of the OVATE gene family may be involved in regulating specific development of *sorghum* leaves and seeds. This study improves the understanding of the OVATE gene family of *sorghum* and provides important clues for further exploration of the function of the OVATE gene family**.**

## Introduction

For many food and industrial crops, the growth and development of grains and seeds are of special importance for their economic value and food security. *Sorghum bicolor* (L.), as an important food and bioenergy crop, is widely planted in many areas around the world^1^. The harvested *sorghum* seeds and stalks can be used not only for brewing wine and sugar, but also as animal feed to improve the industrial value of *sorghum*^2^. Meanwhile, due to its excellent drought resistance and salinity tolerance, *sorghum* has become a representative of C4 model crops and has been applied in many stress tolerance studies ^3^. With the rapid development of the brewing and clean bioenergy industries, the market demand for *sorghum* is gradually increasing. However, compared with field crops such as maize and rice, the study of seed development and yield traits of *sorghum* still needs to be strengthened^4–7^.

Advances in sequencing technology have greatly contributed to the development of plant science, and s*orghum* research has benefited from it no exception^8,9^. In 2009, Paterson et al.^10^ assembled the first sorghum genome with a size of about 730 Mb, and found that gene loss occurred after ancient polyploidy. After that, several *sorghum* genomes of different germplasm were published one after another and in-depth research was carried out at the population level based on these genomes^11–13^. For example, Morris et al.^14^ analyzed the population structure of *sorghum* based on more than 260,000 SNPs identified from 971 germplasm, and revealed the potential genetic loci regulating important agronomic traits through genome-wide association; Tao et al.^15^ constructed the first *sorghum* pan-genome map by assembling and integrating 16 *sorghum* genome data and discovered a large number of presence/absence variants involved in *sorghum* domestication and improvement. The publication of these basic data provides a strong support for further study on stress resistance and quality traits of *sorghum*. At present, some studies focus on the analysis of the stress resistance mechanism of *sorghum* and the identification of seed development regulatory loci. For example, Calone et al.^16^ studied the mechanism of salt tolerance and Na in *sorghum* through experiments; Zhou et al.^17^ revealed the effects of different nitrogen application rates and planting densities on *sorghum* grain yield and quality and Ahn et al.^1^ identified some SNP loci that regulate the morphological development of *sorghum* seeds through genome-wide association analysis.

The OVATE gene family (OFP), as a new type of plant transcription factor first discovered in tomato, has been proved to have various functions such as regulating plant organ development and participating in plant resistance to stress^18,19^. Studies in peppers show that *CaOFP20* can regulate the shape of peppers as an inhibitor of fruit elongation and An et al.'s research indicates that members of ovate family may be the potential regulatory factors for the difference of tea leaf area among different tea varieties^18,20^; Zhou and Guan et al.^21,22^ respectively found that an inversion about 1.7 Mb in length downstream of OVATE family members in peach trees can regulate the shape of peach fruits. In addition, studies in rice and *Arabidopsis* have proved that *OsOFP6* and *AtOFP8* can resist drought and freezing stress by regulating the level of H_2_O_2_ in plants or wax synthesis on the surface of leaves^23,24^. At the same time, Li and Hackbusch found that that members of the OVATE gene family can regulate plant development by combining with TALE or KNOX II family proteins to form complexes^25,26^. In mango, it was found that 25 OVATE gene family members were mainly highly expressed in flowers and immature fruits, and six family members associated with the shape of mango fruit were identified^27^; While most of the OVATE family members in cotton have no introns and play an important role in fiber and ovule development^28^. At present, there are relatively few studies on the OVATE family and most of them focus on the research of its regulatory mechanism for tissue development. The microarray expression analysis shows that members of the OVATE family may be involved in salt tolerance, drought resistance and pest resistance^29^. The above results indicate that the OVATE gene family plays an important role in the process of plant growth and development. However, the number and whether it has the function of regulating tissue development in *sorghum* remain unclear.

In the current study, we have comprehensively identified the members of the OVATE gene family in *sorghum*, and analyzed their gene structure, protein motif and cis-acting elements in the promoter region. Meanwhile, its evolutionary characteristics were analyzed by phylogenetic and genome-wide synteny analysis. On this basis, transcriptome sequencing and RT-qPCR analysis were used to determine the expression levels of the OVATE gene family members in sorghum to reveal the candidate family members that have the potential to regulate tissue development. Our research not only provides a global perspective for the functional evolution of OVATE gene family in *sorghum* genome, but also provides important clues for further understanding its regulatory role in *sorghum* growth and development through quantitative expression analysis.

## Materials and Methods

### Plant materials

The “JingDuXiaoBaiRen” *sorghum* variety is planted in the Moutai College Germplasm Resource Garden in Guizhou Province, China (27.74°N, 106.33°E). Three biological replicates of seeds (S1 and S2 represent seed formation and filling stages, respectively) and leaves (L1 and L2 sampling times correspond to seeds) samples at different developmental stages were collected. Subsequently, these samples are placed in a -80 ℃ refrigerator until they are used.

### Transcriptome sequencing and differentially expressed gene analysis

Prior to transcriptome sequencing, the total RNA of all samples was extracted using the Total RNA Purification Kit (cat DP441, Tiangen, China) according to the manufacturer's protocol. Qualified RNA samples were used for sequencing, and the average sequencing depth was more than 20 × . At the same time, the longest CDS sequence of each gene in the reference genome BTx623 was extracted according to the gff file and indexed using SMALT software^30^. After that, the following parameters of the SMALT software are used to align clean reads with the reference database: -m 35 -j 20. After calculating the gene expression level by selectSmart.pl script, the R package DEGseq was used for the analysis of differentially expressed genes (fold_change > 2 and the q-value < 0.05)^31^. In addition, expression level heatmaps of OVATE gene family members were visualized by TBtools software.

### Identification of OVATE Gene Family in *Sorghum*

BTx623 (Sorghum_bicolor_NCBIv3) and Rio (Sorghum_rio.JGI-v2.0) *sorghum* genome and protein sequence files were downloaded from SORGHUMBASE (https://www.sorghumbase.org/) and used as reference sequences, while the gff files corresponding to the two genomes were also downloaded at the same time^32^. To identify the *sorghum* OVATE gene family, the HMM (The Hidden Markov Model) model was first downloaded from the Protein Family Database (Pfam) (http://pfam.sanger.ac.uk/) website. The HMMsearch software was then used to identify members of the *sorghum* gene family. The specific identification method refers to our previous research^18^. At the same time, in order to ensure the reliability of the identification results, the OVATE sequence of *Arabidopsis thaliana* was downloaded and aligned with the candidate OVATE sequence of sorghum, and the sequence with a coincidence rate of more than 75% was finally retained.

### Chromosome distribution and synteny analysis

After obtaining the location information of OVATE gene family members from the gff file and calculating the length of different chromosomes, the MG2C website (http://mg2c.iask.in/mg2c_v2.1/) was used to visualized the chromosome distribution of the OVATE gene family. After downloading *Arabidopsis* (https://www.arabidopsis.org/) and *maize* (https://maizegdb.org/) genomes, gene duplication and synteny analysis were performed with reference to previous studies^20,33,34^. According to the above results, the collinearity gene pairs were extracted, and the CDS and protein sequences of the OVATE gene family were obtained at the same time, and then the Ka/Ks values were calculated under the default parameters using the Simple Ka/Ks Calculator tool built into TBtools.

### Sequence analysis and phylogenetic tree construction

The MW (molecular weight) and pI (isoelectric point) of the members of the OVATE gene family in sorghum were analyzed by ExPasy site (https://web.expasy.org/). The cis-acting elements were analyzed based on the PlantCARE (https://bioinformatics.psb.ugent.be/webtools/plantcare/html/) online analysis tool, and subcellular localization results for all OVATE gene family members were predicted based on Cell-PLoc 2.0 (http://www.csbio.sjtu.edu.cn/bioinf/Cell-PLoc-2/). At the same time, the built-in MEME program of TBtools is used for motifs analysis. Finally, information such as motifs, cis-acting elements and gene structure (based on the gff file) are displayed graphically through TBtools^34^. The downloaded protein sequences of the OVATE gene family from maize and Arabidopsis were aligned with the Sorghum BTx623 and Rio OVATE gene family sequences using the ClustalW software integrated into MEGA 11. The NJ phylogenetic tree is then constructed with a bootstrap value of 1000^35^.

### Verification of expression levels of OVATE gene family members by RT-qPCR

For RT-qPCR, first-strand cDNA was synthesized from total RNA with the PrimeScript RT Reagent Kit (cat RR036A, Takara, Japan) using the manufacturer’s protocols. A 10 μL RT-qPCR amplification system was performed with the following parameters: 95 °C predenaturation for 3 min, 95 °C denaturation for 10 s, 64 °C annealing for 30 s, for a total of 45 cycles. At the same time, each biological replicate included three technical replicates, and EIF4α was used as the internal reference gene^36^. The relative gene expression values were analyzed using the 2^-△Ct^ method. All primer sequences are listed in Table S1. All experimental methods were carried out in accordance with the relevant guidelines and regulations.

### Data analysis

The data were analyzed for data processing and significant differences using SPSS 22.0 and plotted using Python 3.6 and TBtools. The data obtained were shown below mean ± standard deviation (SD). Data were all analyzed using Student’s t-test and one-way ANOVA. In the representation of the results, different letters were used to indicate that the differences were statistically significant (P < 0.05).

## Results

### Identification of OVATE Gene Family in *Sorghum*

**Figure 1:**
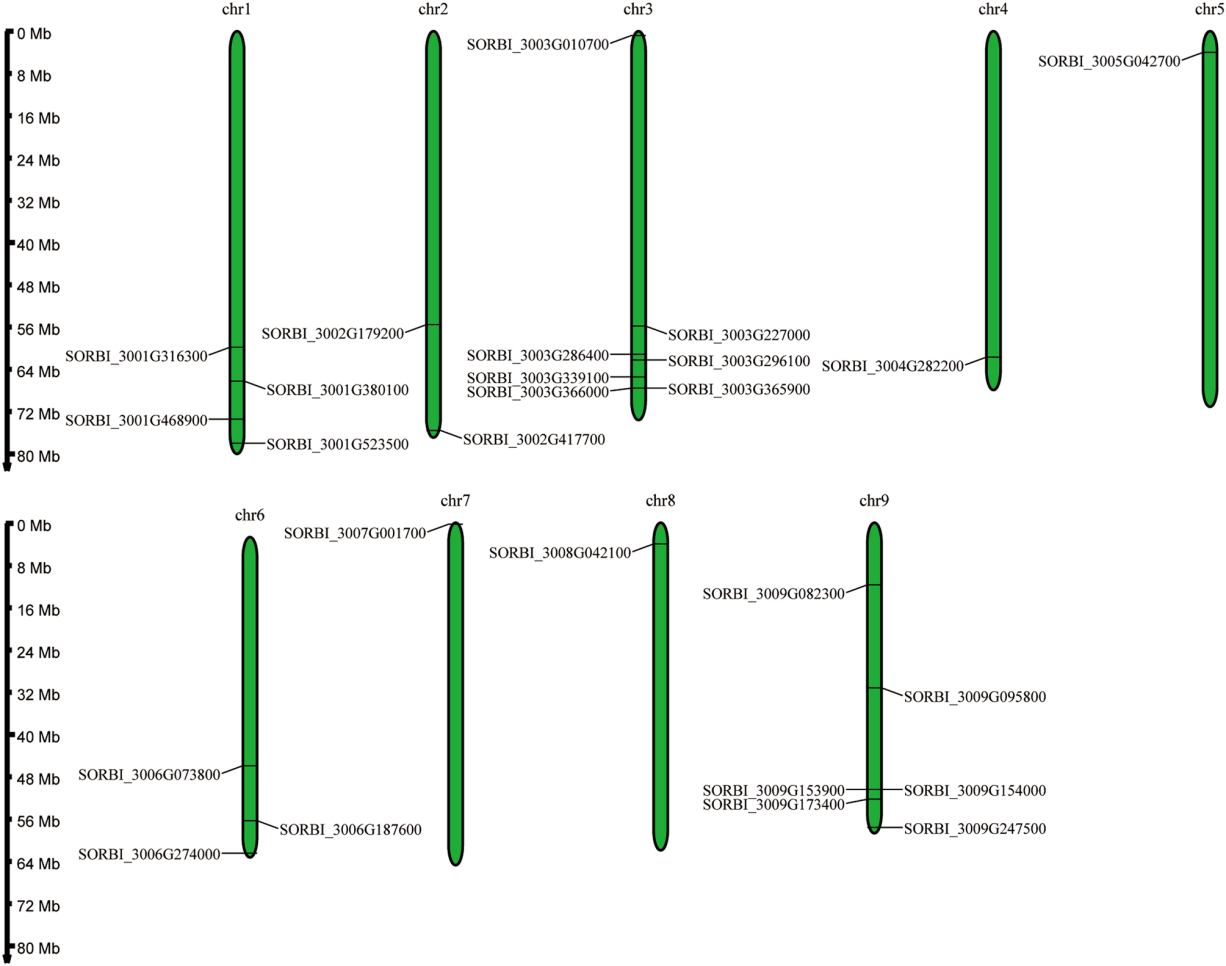
Distribution of the OVATE genes in the BTx623 genome. The left axis shows the length of each chromosome, and it was estimated in mega base (Mb).

A total of 26 OVATE gene family members were identified from the *sorghum* genome (BTx623), which were unevenly distributed on all chromosomes except chromosome 10 (Fig. 1). Among them, chromosomes 4, 5, 7 and 8 each contain an OVATE gene. For the remaining chromosomes, the number of OVATE family members ranged from 2 (chromosome 2) to 7 (chromosome 3). The sequence analysis showed that the protein length of OVATE gene family ranged from 229 amino acids (*SORBI_3009G153900*) to 543 amino acids (*SORBI_3003G339100*), corresponding to the minimum 24.68 kDa and the maximum 60.06 kDa of OVATE family members (with an average of 35.63 kDa) (Table S2). Isoelectric point calculations found that the pI values of different OVATE proteins varied between 4.49 (*SORBI_3005G042700*) and 11.14 (*SORBI_3003G296100*) with an average of 8.64. At the same time, bioinformatics prediction results show that most OVATE genes are located in nucleus, but some members are also predicted to be located in cell membrane (*SORBI_3003G365900* and *SORBI_3005G042700*) and chloroplast (*SORBI_3001G316300* and *SORBI_3001G523500*) (Table S2). The differences in MW and pI indicate that these genes may have functional differentiation in *sorghum*.

### Phylogenetic analysis of the OVATE gene family

**Figure 2:**
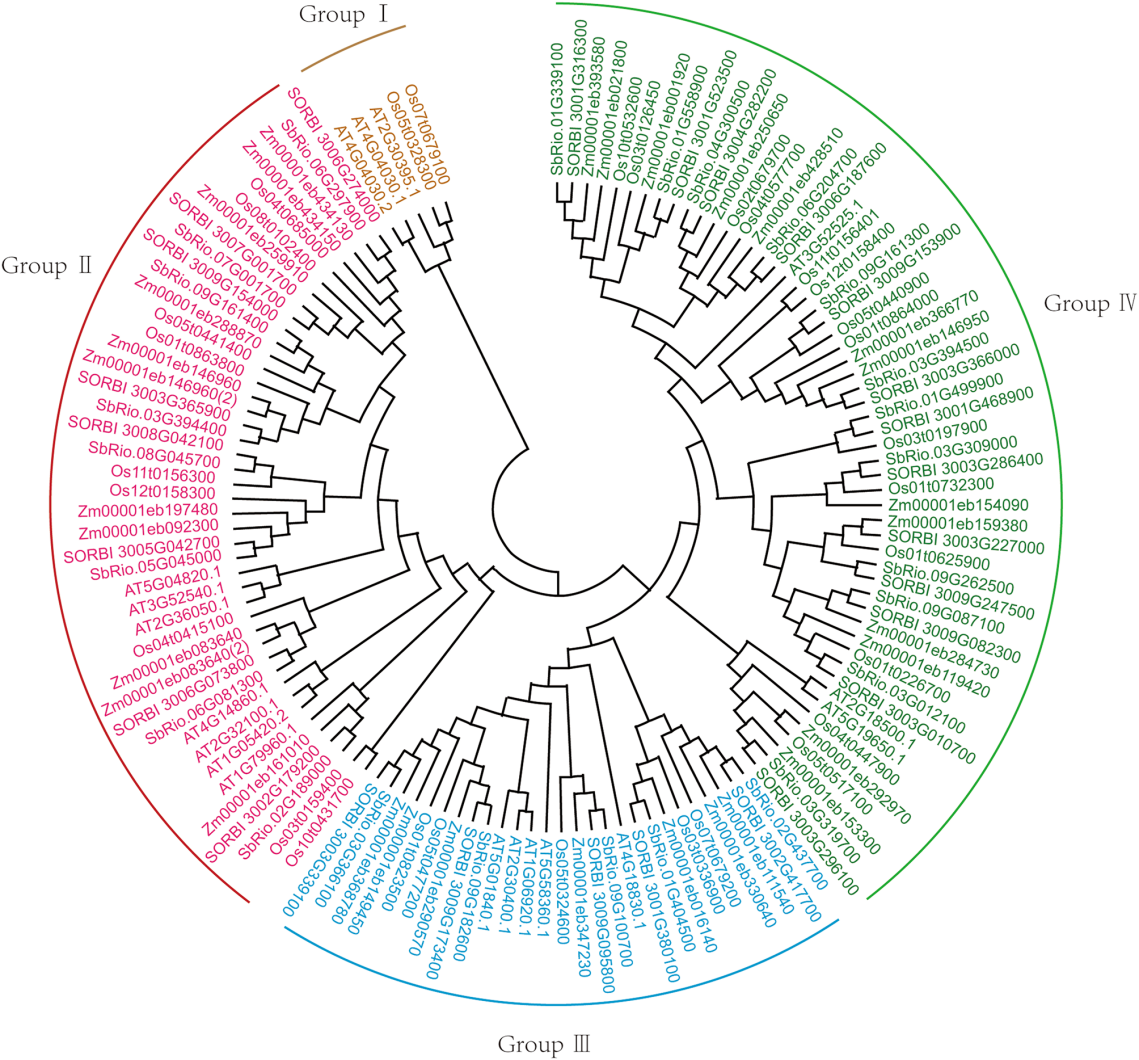
Phylogenetic analysis of OVATE genes in *sorghum*. An unrooted Neighbor–Joining phylogenetic tree was constructed from *Arabidopsis*, *Maize*, *BTx623* and *Rio*. The bootstrap test was set to 1,000 replicates.

In order to reveal the evolutionary relationship between the *sorghum* OVATE gene family and other species, the OVATE proteins of rice, maize, *Arabidopsis* and sweet *sorghum* varieties Rio were collected together with the sequences identified in this study to construct phylogenetic trees (Table S3). The results are shown in Fig. 2. All sequences were divided into four groups, of which Group contained only rice and *Arabidopsis* OVATE proteins. In the Group II, there were 8 members of the OVATE family from BTx623 and 8 from the Rio genome, while in the Group III, there were 5 OVATE family members from both *sorghum* genomes. The Group IV contained the largest number of sorghum OVATE gene family members, reaching 25, of which 13 were from BTx623 and 12 from Rio. The highly similar clustering results showed that there was no significant differentiation of the OVATE gene family in grain *sorghum* and sweet *sorghum*.

### Protein motif, *cis*-acting elements and gene structure analysis

**Figure 3:**
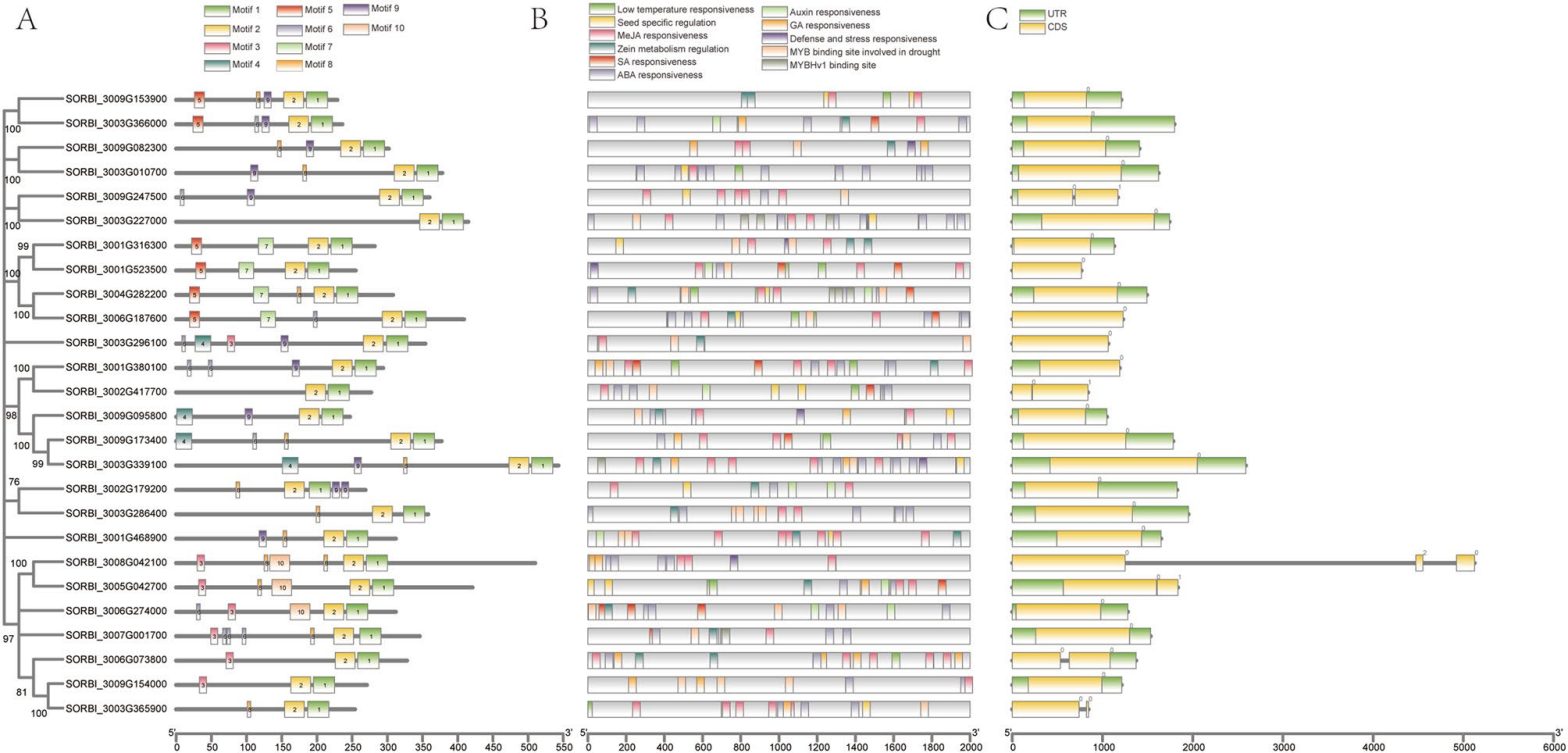
Motif, cis-acting regulatory elements and gene structure analysis of the OVATE gene family. (**A**) Protein motifs of OVATE genes. Ten conservative motifs are shown in the figure. (**B**) Cis-acting regulatory elements analysis of the OVATE genes. Sequences of the 2000 bp above the start codon were used to identify cis-acting elements. (**C**) Gene structure of OVATE genes. The yellow and green blocks indicate CDS and UTR respectively, and the lines represent introns. The lengths of blocks and lines represent relative sequence lengths.

We analyzed the protein-conserved motifs of all 26 members of the OVATE gene family. The result is shown in Fig. 3A. A total of 10 conserved motifs were identified, with each protein sequence containing 3 to 7 motifs. Among them, motif 1 and motif 2 were the most conserved and identified in all protein sequences, while *SORBI_3003G296100* contained the most motif types. Since cis-acting elements play an important role in gene transcription and expression, we performed cis-acting element analysis on the first 2000 bp sequence of promoters of OVATE family members (Fig. 3B). Interestingly, we found a large number of hormone-related cis-acting elements, including GA, MeJA, ABA and SA response elements. At the same time, some cis-acting elements related to drought and low temperature stress and involved in the regulation of seed development were identified. These results suggest that members of the OVATE gene family of *sorghum* are not only able to participate in resisting various environmental stresses, but may also regulate the development of organs such as *sorghum* seeds. At the same time, it should be noted that a large number of light-responsive elements are also found in the promoter region. Gene structure analysis showed that except for the gene *SORBI_3008G042100* containing 3 exons, other members of the OAVET family contained 1 to 2 exons (Fig. 3C).

### Segment duplication and collinearity analysis

**Figure 4:**
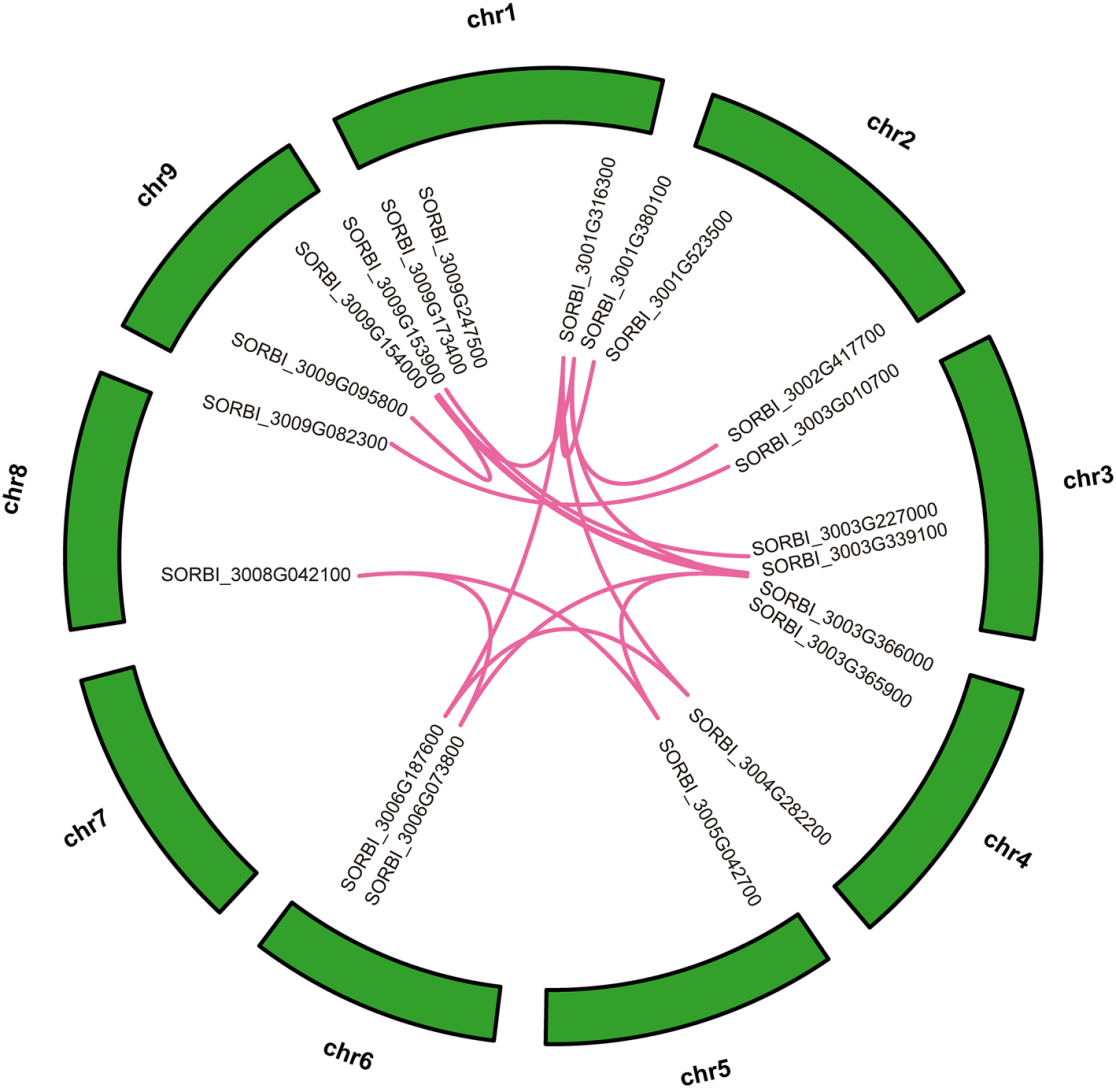
Synteny analysis of OVATE genes in BTx623 genome. The red line is a large segment replication between gene family members.

Many studies have shown that segment duplication may drive the expansion of gene families in the plant genome^33,37^. Therefore, to explore the mechanism of expansion of the OVATE gene family in *sorghum*, we performed segment duplication event analysis. As shown in Fig. 4, the duplication event exists widely on 9 chromosomes. Twenty genes from 26 OVATE family members participated in the formation of 19 gene pairs (Table S4). Among them, some genes such as *SORBI_3001G380100* formed multiple gene pairs. Most gene pairs are located on different chromosomes, which indicates that segment duplication is an important driving force for the expansion of sorghum OVATE gene family. However, in the process of expansion, their evolutionary direction is still unknown. In order to determine the effect of selection pressure on these genes, we calculated the Ka/Ks values of all homologous genes (Table S5). The results show that all Ka/Ks values are less than 1, which indicates that they have experienced a strong purifying or negative selection^35^.

**Figure 5:**
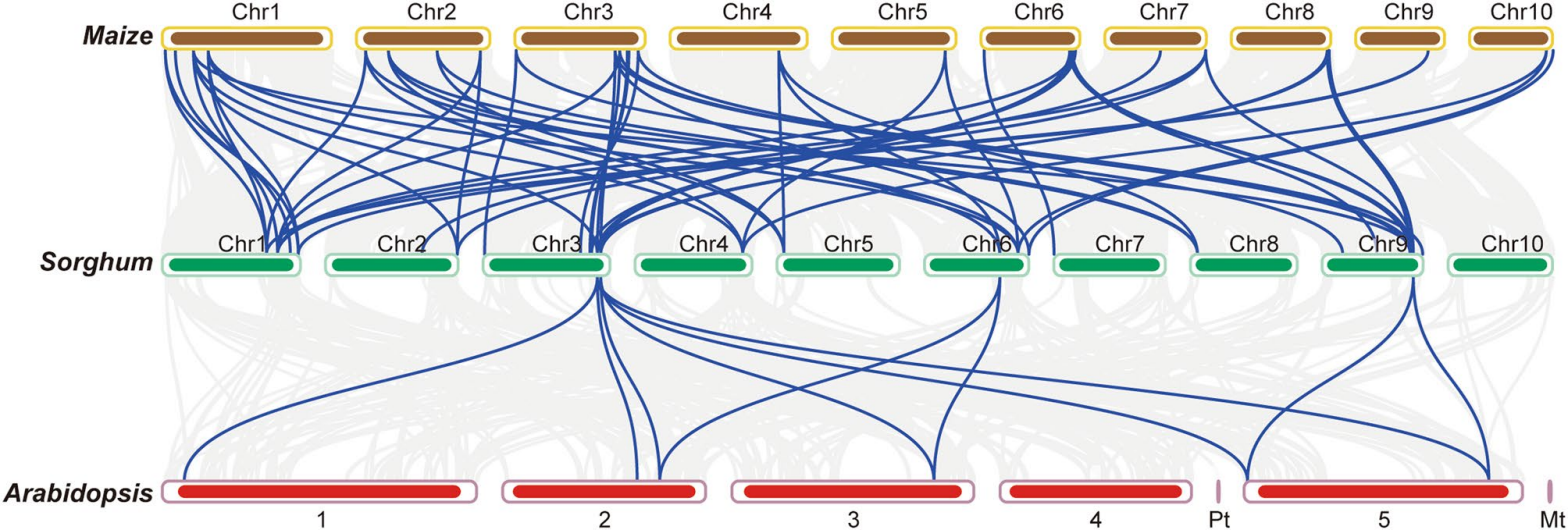
Synteny analysis of OVATE genes from Sorghum, Maize and Arabidopsis.

Furthermore, we performed collinearity analysis to reveal the origin and evolutionary relationship of OVATE gene family in different species (Fig. 5). The results showed that 63 homologous gene pairs were identified between *sorghum* and *maize* genomes, and some *sorghum* genes were found to participate in the formation of gene pairs many times. However, only ten gene pairs of collinearity were found between *sorghum* and *Arabidopsis* genome. These results suggest that there is a closer genetic relationship between *sorghum* and *maize*, and some members of OVATE family have expanded in *maize*.

### Analysis of OVATE gene expression using transcriptome data

**Figure 6:**
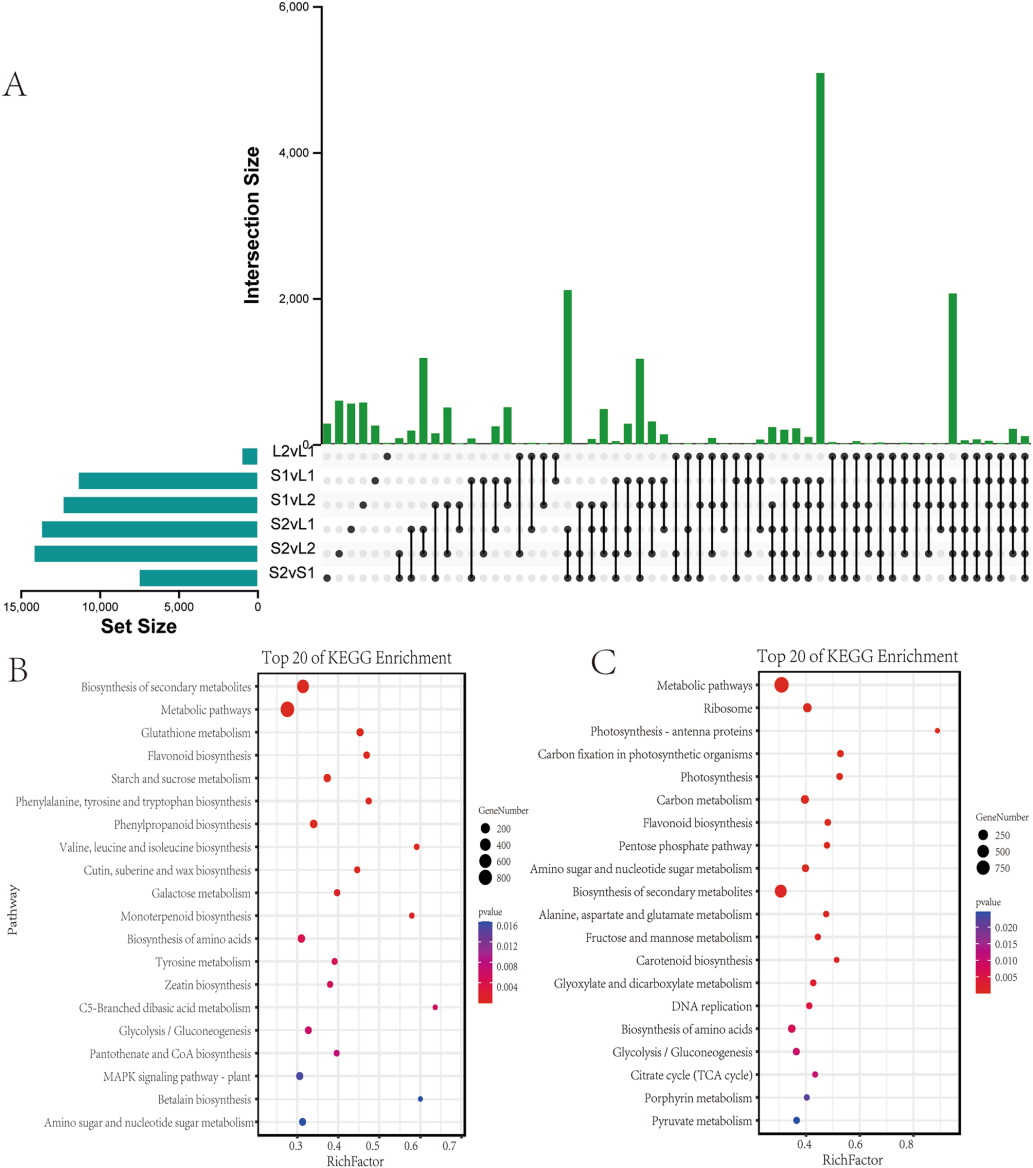
Differential expression of genes and KEGG enrichment analysis in *sorghum* seeds and leaves. (**A**) Upset plot of differentially expressed genes at different developmental stages of *sorghum* seeds and leaves. (**B**) KEGG enrichment analysis of S2vS1 differentially expressed gene. (**C**) KEGG enrichment analysis of differentially expressed genes between leaves and seeds (S1vL1, S1vL2, S2vL1, S2vL2).

In order to reveal the expression characteristics of OVATE gene family in *sorghum*, we selected leaves and seeds of three different development stages for transcriptome sequencing. Transcriptome analysis showed that there were a large number of differentially expressed genes in different tissues (Fig. 6). As shown in Fig. 6A, there are the most differentially expressed genes in the two developmental stages of S2 and L2, with a number of 14,116, and the differentially expressed genes in different developmental stages of leaf are only 940 (L2vL1); between leaves and seeds, the differentially expressed genes shared by different developmental stages reached more than 5000. Meantime, the differentially expressed genes in different stages of seed development reached 7445 (S2vS1), which was much higher than that in leaves (L2vL1). In order to further explore the functional characteristics of the difference genes, we performed a KEGG enrichment analysis of the difference genes between seeds and leaves. The results showed that for S1vS2, the differential genes were mainly enriched in the biosynthesis of secondary metabolites such as flavonoids and polyphenols (Fig. 6B). However, between seeds and leaves, a large number of differential genes are enriched not only in metabolism-related pathways, but also in carbon metabolism and photosynthesis-related pathways (Fig. 6C).

**Figure 7:**
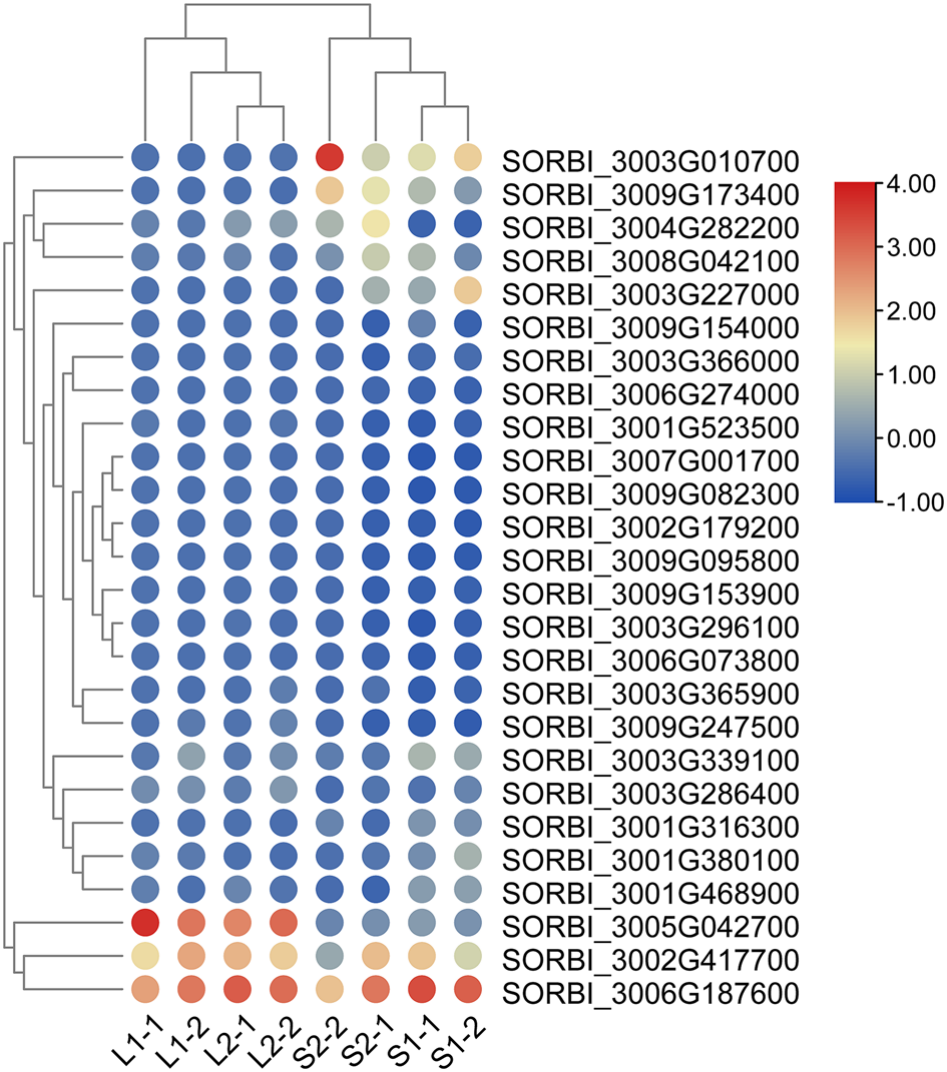
The expression pattern of the OVATE gene family in *sorghum* seeds and leaves was analyzed by heat map.

Furthermore, based on the transcriptome sequencing data, we analyzed the expression levels of OVATE gene family members in different tissues. The results are shown in Fig. 7, most members of the OVATE gene family did not have different expression in *sorghum* seeds and leaves, while some genes such as *SORBI_3003G010700* and *SORBI_3009G173400* were expressed at higher levels in *sorghum* seeds, but some genes were expressed at significantly higher levels in leaves than seeds (such as *SORBI_3005G042700*).

### Verification of expression of members of the OVATE gene family of *sorghum* by RT-qPCR

**Figure 8:**
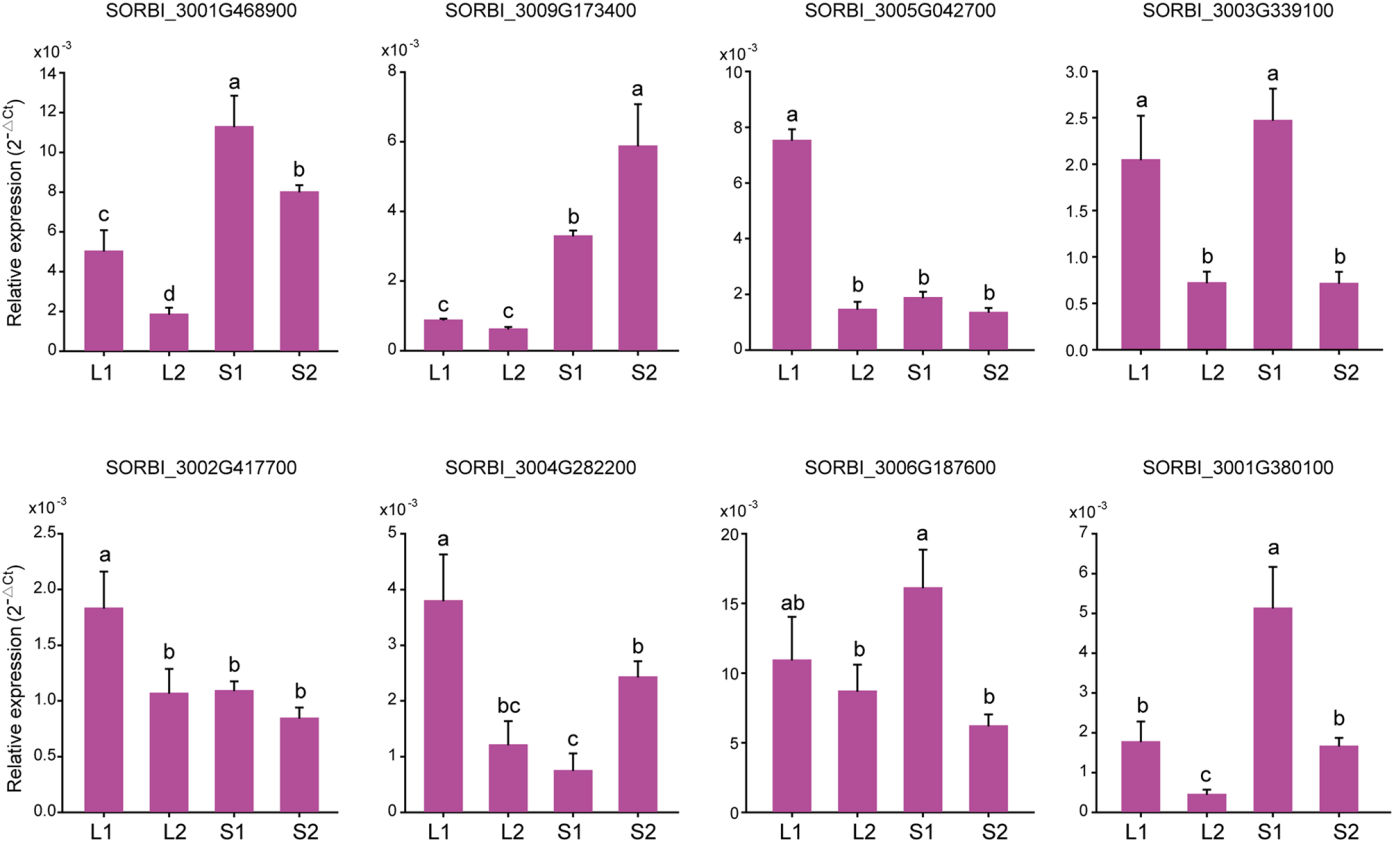
Expression of OVATE genes analyzed by RT-qPCR in different tissues of *sorghum*. Different letters above the bars represent significant differences at p < 0.05. The bars are standard deviations (SD) of three biological replicates.

To verify the expression levels of OVATE gene family members in *sorghum* seeds and leaves, we designed 26 pairs of primers for RT-qPCR amplification. Due to the imbalance in sequence GC content and the presence of repetitive sequences, only 8 genes were successfully validated in the end (some family members do not express or have extremely low expression levels and are difficult to be verified) (Fig. 7). The results showed that these genes had multiple expression patterns in the process of *sorghum* tissue development (Fig. 8). For example, except for *SORBI_3003G339100* and *SORBl_3006G187600*, the expression levels of all genes in S1 and L1 are significantly different. Among them, with the development of leaves and seeds, the expression level of some genes gradually decreased (*SORBI_3002G417700* and *SORBl_3005G042700*). This indicates that members of OVATE gene family may be involved in regulating the development of *sorghum* leaves and seeds. At the same time, the similar expression level of RT-qPCR and transcriptome proves the reliability of the above results.

## Discussion

Exploring the growth and development of seeds plays an important role in improving the yield and economic value of *sorghum*. In previous studies, some researchers have conducted in-depth research on the development regulation of *sorghum* seeds. For example, Tao et al.^38^ identified 114 candidate genes associated with *sorghum* seed size, of which 63 showed a signal of purification selection during domestication; Zhang et al.^39^ identified 73 QTLs related to grain color and tannin content in China *sorghum* materials through genome-wide association study, and found a new recessive allelic variant of *TAN2* gene. Many studies in other plants show that OVATE gene family plays an important role in regulating the development of plant organs and resisting stress. Studies have shown that *OsOFP6* in rice not only participates in the growth regulation of rice plant development, but also may enhance the drought tolerance of rice^40^; In tomato, OVATE can not only regulate the shape of fruit, but also affect the development of flower organs and pollen by regulating the signal transduction of BR and GA^41^. However, the biological function of OVATE gene family in sorghum has not been revealed. In this study, we identified 26 OVATE gene family members from the sorghum genome, which are more numerous than *Arabidopsis thaliana* but lower than rice, and equal to the number of OVATE gene family members in tea plants^18,42,43^. These genes are unevenly distributed across nine chromosomes, with numbers ranging from 1 (chr4, chr5, chr7, chr8) to 7 (chr3). Phylogenetic analysis showed that 26 OVATE genes were classified into four groups, and OVATE gene family members showed high homology in grain *sorghum* and sweet, suggesting that the OVATE gene family had a low degree of differentiation among different *sorghum* lines.

The motif analysis found that motif 1 and motif 2 were identified in all members of the OVATE gene family, but some specific motifs were only identified in some genes. For example, motif 5 and motif 7 are only found in the four genes of *SORBI_3001G316300*, *SORBI_3001G523500*, *SORBI_3004G282200* and *SORBI_3006G187600*, while motif 10 is only identified in *SORBI_3008G042100*, *SORBI_3005G042700* and *SORBI_3006G274000*. These findings suggest that these motifs may be important for functional diversity differentiation of the OVATE gene in *sorghum*^20,37^. In addition to the common light-responsive elements, a large number of hormone-related cis-acting elements were found in the promoter sequence of 2000 bp upstream of the OVATE genes. At the same time, in addition to some cis-acting elements associated with abiotic stresses such as low temperature and drought, seed-specific regulation related elements were also identified. These results suggest that members of the OVATE gene family in *sorghum* may be responsible for multiple biological functions.

Segmental and tandem duplication are important drivers of gene family expansion^44^. Evolutionary analysis within the genome showed that members of the OVATE gene family formed 19 gene pairs, some of which were involved in the formation of gene pairs multiple times. These results suggest that segmental duplication events play an important role in the expansion of the OVATE gene family in *sorghum*^45,46^. Collinear analysis showed more collinear gene pairs between maize and sorghum compared to *Arabidopsis*, suggesting that the expansion of the OVATE gene family may have occurred after the differentiation of sorghum and *Arabidopsis*. In addition, Ka/Ks analysis showed that members of the OVATE gene family had a higher frequency of harmful mutations, and they were in the state of purification or negative selection^35,42,47^.

To explore the expression characteristics of OVATE gene family members during *sorghum* leaf and seed development, we performed transcriptome sequencing of leaves and seeds in two developmental stages. The results of transcriptome analysis showed that there were a large number of differentially expressed genes between sorghum leaves and seeds at different stages of development. For example, more than 5,000 differential genes are shared between S1vL1, S1vL2, S2vL1, and S2vL2. KEGG analysis showed that metabolite-related pathways were enriched at different stages of seed development and between seed and leaf. Furthermore, we focused on the expression analysis of members in the OVATE gene family in different tissues of *sorghum*. The results showed that members of the OVATE gene family exhibited diverse expression characteristics during the development of leaves and seeds, suggesting their potential for diversified regulatory functions^19,29,48^. It needs to be pointed out that the transcriptome analysis results show that some members of the OVATE family do not express or have extremely low expression levels in *sorghum*, which indicates that these genes may not undertake regulatory functions in *sorghum* leaves and seeds. In addition, due to a large number of repetitive sequences, it is very difficult to design appropriate primer pairs. Finally, only the expression levels of 8 OVATE gene members were successfully verified by RT-qPCR analysis, proving that the expression levels of these genes are tissue-specific. For example, *SORBl_3001G468900*, *SORBI_3009G173400* and *SORBl_3001G380100* are expressed significantly higher in seeds than in leaves, while gene *SORBl_3005G042700* and *SORBl_3004G282200* are highly expressed in leaves (L1 stage). Meantime, the *SORBI_3002G417700*, *SORBI_3001G380100*, *SORBI_3009G173400*, *SORBI_3003G339100* and *OFP4* (*AT1G06920*) in *Arabidopsis thaliana* are highly homologous, and they are all clustered in Group III, while the latter has been confirmed to have the function of regulating cell wall formation^25^. Homology analysis found that the genes *SORBI3009G173400* and *SORBI3003G339100* in *sorghum* have relatively high homology with *SlOFP20* in tomatoes that regulates floral organ and pollen development^41^. These results strongly suggest that the eight genes verified by RT-qPCR may play an important role in the development process of *sorghum* tissues and organs.

## Conclusions

In this study, we performed a comprehensive identification of the OVATE gene family based on the BTx623 *sorghum* genome. The 26 OVATE gene family members are spread across 9 chromosomes and are divided into four groups. Promoter sequence analysis showed that a large number of hormone-related cis-acting elements were identified, suggesting that members of the OVATE gene family may be involved in the development process of *sorghum* tissues and organs. At the same time, evolutionary analysis showed that the OVATE family members in *sorghum* had expanded after differentiation with *Arabidopsis*, and all members were subject to purification selection. RNA sequencing and RT-qPCR analysis showed that members of the OVATE gene family exhibited diverse expression characteristics in sorghum leaves and seeds. These results will provide fundamental support for our deeper understanding of the biological function of the OVATE gene family in *sorghum* and promote the *sorghum* breeding process.

### Supplementary Information

Supplementary Table S1.

Supplementary Table S2.

Supplementary Table S3.

Supplementary Table S4.

Supplementary Table S5.

## Data Availability

Transcriptome sequencing data was uploaded to the National Bioinformatics Center of China (CNCB), the BioProject accession number is PRJCA020623 (https://ngdc.cncb.ac.cn/gsa/browse/CRA013087).
